# Collagen turnover is associated with cardiovascular autonomic and peripheral neuropathy in type 1 diabetes: novel pathophysiological mechanism?

**DOI:** 10.1186/s12933-023-01891-8

**Published:** 2023-06-29

**Authors:** Christian S. Hansen, Daniel G. K. Rasmussen, Tine W. Hansen, Signe Holm Nielsen, Simone Theilade, Morten A. Karsdal, Federica Genovese, Peter Rossing

**Affiliations:** 1 Steno Diabetes Center Copenhagen, Niels Steensens Vej 2-4, 2820 Gentofte, Denmark; 2 Nordic Bioscience, Herlev, Denmark; 3 Technical University of Denmark, Lyngby, Denmark; 4 Department of Clinical Medicine, University of Copenhagen, Copenhagen, Denmark

**Keywords:** Cardiovascular autonomic neuropathy, Collagen, Diabetes, Inflammation, Peripheral neuropathy, Cardiovascular disease

## Abstract

**Background:**

Diabetic cardiovascular autonomic neuropathy (CAN) and distal symmetrical polyneuropathy (DSPN) are severe diabetic complications. Collagen type VI (COL6) and III (COL3) have been associated with nerve function. We investigated if markers of COL6 formation (PRO-C6) and COL3 degradation (C3M) were associated with neuropathy in people with type 1 diabetes (T1D).

**Methods:**

In a cross-sectional study including 300 people with T1D, serum and urine PRO-C6 and C3M were obtained. CAN was assessed by cardiovascular reflex tests: heart rate response to deep breathing (E/I ratio), to standing (30/15 ratio) and to the Valsalva maneuver (VM). Two or three pathological CARTs constituted CAN. DSPN was assessed by biothesiometry. Symmetrical vibration sensation threshold above 25 V constituted DSPN.

**Results:**

Participants were (mean (SD)) 55.7 (9.3) years, 51% were males, diabetes duration was 40.0 (8.9) years, HbA_1c_ was 63 (11 mmol/mol, (median (IQR)) serum PRO-C6 was 7.8 (6.2;11.0) ng/ml and C3M 8.3 (7.1;10.0) ng/ml. CAN and DSPN were diagnosed in 34% and 43% of participants, respectively. In models adjusted for relevant confounders a doubling of serum PRO-C6, was significantly associated with odds ratio > 2 for CAN and > 1 for DSPN, respectively. Significance was retained after additional adjustments for eGFR only for CAN. Higher serum C3M was associated with presence of CAN, but not after adjustment for eGFR. C3M was not associated with DSPN. Urine PRO-C6 analyses indicated similar associations.

**Conclusions:**

Results show previously undescribed associations between markers of collagen turnover and risk of CAN and to a lesser degree DSPN in T1D.

## Background

Neuropathy in diabetes is a common and severe complication to the disease, which can affect both autonomic and sensory nerves. Autonomic neuropathy is often manifested as a dysfunction of the autonomic nervous system regulating the heart rate and vessel tone, known as cardiovascular autonomic neuropathy (CAN). The prevalence of CAN ranges from 20% in unselected populations with diabetes [1, 2] to 35–65% in people with long-standing diabetes [3]. CAN is an independent risk factor for cardiovascular mortality and morbidity [4–7], diabetic nephropathy [8] and disorders of bone metabolism [9]. For decades strides have been taken to compose disease-modifying treatment for CAN. However, no treatment is available. Distal symmetrical polyneuropathy (DSPN) results from damage to the peripheral nervous system and is often presented as loss of sensation and/or pain of the distal limbs. DSPN is a highly prevalent complication of both type 1 and type 2 diabetes. Reported prevalences of DSPN vary. Estimates suggests that 20% of people with type 1 diabetes have signs of DSPN 20 years after diabetes diagnosis [10, 11]. No disease-modifying treatment is available for DSPN. Common pathogenic pathways lead to CAN and DSPN, including inflammation, reactive oxygen species, and gluco- and lipotoxicity [12, 13]. Identification of new risk factors may expose new means of intervention for CAN and DSPN. Higher levels of markers of collagen turnover such as serum PRO-C6 (marker of collagen type VI (COL6) formation) and C3M (marker of collagen type III (COL3) degradation) have been associated with diabetic complications, such as diabetic nephropathy and mortality [14–17]. In addition, higher levels of collagen turnover markers have been associated with impaired nerve function in animal models [18, 19] and people with diabetes [20–22]. The association between markers of collagen turnover and neuropathy may reflect an increased accumulation of extracellular matrix around the nerves and possibly the pro-inflammatory and pro-fibrotic effect that some by-product of collagens acquire as seen for the COL6-derived bioactive fragment endotrophin [23]. In concert, collagen turnover and thereby fibrosis and inflammation caused by collagen signaling may be part of the pathophysiological mechanisms leading to diabetic neuropathy and could therefore be a future treatment target.

We investigated the possible associations between serum and urine PRO-C6 and C3M and presence of diabetic neuropathy in people with type 1 diabetes (T1D). Primary outcome was the CAN diagnosis and secondary outcome were continuous measures of cardiovascular autonomic reflex tests used to diagnose CAN and lastly the DSPN diagnosis.

## Methods

### Study design

Participants in this cross-sectional study were people with T1D following the standard medical care at the outpatient clinic of Steno Diabetes Center Copenhagen, Denmark from 2009 to 2011 as described previously [24]. The study cohort consisted of 355 participants. Twenty-six participants were outside the age range of 20 to 80 years and were not included in the analyses due to non-existing reference values of CAN measures outside this age range. Of the remaining 329 participants, 29 had no data on serum collagen markers or confounders, leaving 300 participants for analyses. Of these participants, 41 participants did not have sufficient autonomic tests performed to assess the CAN diagnosis, leaving 259 participants for analyses. Of the aforementioned 300 participants, all had adequate data on DSPN.

### Assessment of collagen markers

PRO-C6 and C3M were measured in serum (sPRO-C6, sC3M) and urine (uPRO-C6, uC3M) using competitive ELISAs developed by Nordic Bioscience (Herlev, Denmark). The monoclonal antibodies used in the PRO-C6 and C3M ELISAs specifically detect the last 10 amino acids of the COOH-terminal of the a-3 chain of COL6 (^31689^KPGVISVMGT9^3177^) [25] and the neoepitope generated by matrix metalloproteinase-9-mediated cleavage of COL3 (^610^KNGETGPQGP^619^) [26], respectively. Intra- and inter assay variations of the ELISAs were below 10 and 15%, respectively. To normalize for urine output, urinary levels of the markers were normalized to urinary creatinine levels measured using the ADVIA 1800 Chemistry System (Bayer HealthCare). The ELISAs were carried out as previously described [14–16].

### Assessment of autonomic function

Participants rested for 5 min in a supine position in a quiet room at room temperature (18–23 °C) prior to assessment of autonomic function.

Autonomic function was assessed by measures of CAN by 2-min resting heart rate variability (HRV) indices and cardiovascular autonomic reflex test (CARTs). Subsequent to the HRV measurement, the three standard CARTs recommended for diagnosing CAN [27] were performed: the lying-to-standing test (30/15), the deep breathing test (E/I ratio) and the Valsalva maneuver. CARTs were performed in the mentioned order.

Age-dependent cut-off levels defined by Cardone et al. [28] were used to define pathological results of the CARTs. The CAN diagnosis was defined as the presence of two or three pathological CARTs as recommended by the American Diabetes Association [12] and classified for participants with more than one valid CART measure. Participants with one or no CARTs measures were classified as “no CAN estimation”. Higher values imply better autonomic function. Only the CAN diagnosis was used for analyses in the present study.

CAN outcomes were recorded by trained technicians using a Vagus^tm^ device (Medicus Engineering, Aarhus, Denmark).

### DSPN assessment

Vibration perception threshold (VPT) was determined using a Bio-Thesiometer (Bio-medical instruments, Ohio, USA) at the distal end of the great toe on both sides. Participants with a bilateral vibration perception threshold of  ≥ 25 V was considered as having DSPN.

### Biochemical measures

HbA_1c_ was measured by high-performance liquid chromatography (Variant, Bio-Rad Laboratories, Munich, Germany) and serum creatinine concentration by an enzymatic method (Hitachi 912; Roche Diagnostics, Mannheim, Germany). Lipids were analyzed by standardized methods. Urinary albumin excretion ratio was measured in 24-h urine collections by an enzyme immunoassay. Chronic Kidney Disease Epidemiology Collaboration Equation was used to calculate the estimated glomerular filtration rate (eGFR) from plasma creatinine.

### Anthropometric measures

Height and weight were measured with light indoor clothing, without shoes, using a fixed rigid stadiometer (Seca, Chino, USA) and an electronic scale (Mettler Toledo, Glostrup, Denmark), respectively.

### Blood pressure

Oscillometric (A&D Medical, UA787) office blood pressure was measured in a supine position after 15 min rest using an appropriate cuff size. Three measurements were obtained and averaged.

### Lifestyle measures

Lifestyle measures were obtained by questionnaires. Participants were classified as current smokers if using ≥ 1 cigarettes or cigars or pipes per day and all others were classified as non-smokers. Physical activity was defined as being regularly physical active or not.

### Statistical analyses

Participant characteristics are presented as means with standard deviations (SD), as medians with interquartile range (IQR) or as absolute numbers (percentages). Group differences in baseline variables were assessed by linear regression analyses for continuous variables and the chi-square test for categorical variables Linear logistic regression analyses were performed to assess associations between collagen markers as determinants and the binary CAN diagnosis and DSPN diagnosis as outcomes.

Determinant were log 1.5 transformed, thus result after back transformation estimates shown as a function of a 50% increase in collagen markers given in % change. To access associations in participants with normal kidney function, sensitivity analyses, for analyses where the CAN diagnosis was the outcome, were performed for participants with eGFR ≥ 90 ml/min/1.73 m^2^.

Linear regression analyses were performed for analyses of continues outcomes. These outcomes were the CARTs; the lying-to-standing test (30/15), the deep breathing test (E/I ratio) and the Valsalva maneuver (Valsalva ratio). Determinants were log 1.5 transformed and outcomes were log transformed. Regression estimates were back transformed. Thus, the estimates of theses analyses are % change by a doubling of collagen turnover markers.

All analyses were performed using three levels of confounder adjustment: Model 1: adjusted for sex and age; Model 2: as Model 1 and additionally adjusted for HbA_1c_, body mass index, diabetes duration, systolic blood pressure, LDL cholesterol, smoking and beta blocker treatment; Model 3: as Model 2 and additionally adjusted for eGFR. Due to 39 missing values of urinary albumin excretion rate additional adjustments for albuminuria were not performed.

A complete-case analysis approach was used and a two-sided level of significance of 5% was assumed. Analyses were performed using SAS enterprise guide 7.15 (SAS Institute, Cary, NC).

## Results

The total cohort of 300 participants had a mean (SD) age of 56 (9) years, 51% were male, mean diabetes duration was 40.0 (8.9) years, HbA_1c_ was 63 (11) mmol/mol (7.9 (1.0) %), eGFR was 78 (25) ml/min/1.73m^2^, systolic blood pressure 132 (18) mmHg and LDL cholesterol 2.5 (0.7) mmol/mol.

Of the 300 participants included, 171 (57%) had no CAN, 88 (29.3%) had CAN and 41 (13.7%) had no CAN estimation. DSPN was observed in 129 (43%) of participants.

Characteristics for participants stratified by no CAN and CAN (Table 1), show that participants with CAN were marginally older (approx. 1 year), had a longer diabetes duration, poorer metabolic control assessed by HbA_1c_ and numerically higher levels of sPRO-C6 and sC3M than participants without CAN.

**Table 1:** Participant characteristics according to diagnosis of cardiovascular autonomic neuropathy

	No CAN (n = 171)	Definite CAN (n = 88)	P for difference between no CAN and definite CAN	No CAN estimation (n = 41)
Sex (male), (n/%)	78/46	49/56	0.060	25/61
Age (years)	55.4 (9.5)	54.2 (8)	0.572	60.6 (9.6)
HbA_1c_ (mmol/mol)	61.3 (9.6)	66.5 (12.3)	0.008	61.5 (11.5)
HbA_1c_ (%)	7.8 (0.9)	8.2 (1.1)	0.009	7.8 (1.1)
Body mass index (kg/m^2^)	24.9 (3.7)	25 (4.4)	0.857	24.2 (3.2)
Systolic blood pressure (mmHg)	130.7 (17.2)	135.3 (17.5)	0.002	130.3 (17.9)
Diastolic blood pressure (mmHg)	74.0 (8.8)	74.2 (9)	0.843	73.0 (8.5)
Diabetes duration (years)	38.6 (8.6)	41 (8.3)	0.002	44.3 (10.2)
eGFR (ml/min/1.73 m^2^)	84.8 (20.6)	65.1 (27.4)	< 0.001	77.9 (24.4)
Urinary albumin excretion rate (mg/24-h)	9.5 (6;22)	45.5 (14;333)	< 0.002	10.8 (5.8;53.5)
Total cholesterol (mmol/L)	4.7 (0.7)	4.5 (0.9)	0.063	4.6 (0.8)
HDL cholesterol (mmol/L)	1.8 (0.6)	1.7 (0.5)	0.037	1.7 (0.7)
LDL cholesterol (mmol/L)	2.4 (0.6)	2.4 (0.7)	0.444	2.5 (0.7)
Triglycerides (mol/L)	0.9 (0.7;1,2)	1.0 (0.8;1,3)	0.181	0.8 (0.7;1.1)
Betablocker treatment (n/%)	12/7	17/20	< 0.001	7/17
Diuretic treatment (n/%)	66/39	71/81	< 0.001	20/49
RAAS blocker treatment (n/%)	71/42	4/7	< 0.001	15/37
Statin treatment (n/%)	92/54	71/81	0.002	19/46
Pathological E/I ratio (n/%)	27/16	86/98	< 0.001	20/65
Pathological 30/15 ratio (n/%)	30/15	78/89	< 0.001	6/21
Pathological Valsalva (n/%)	4/3	42/78	< 0.001	1/33
E/I ratio	1.2 (1.1;1.3)	1.0 (1.0;1.1)	< 0.001	1.1 (1.1;1.2)
30/15 ratio	1.1 (1.1;1.2)	1.0 (1.0;1.0)	< 0.001	1.1 (1;1.1)
Valsalva ratio	1.5 (1.4;1.8)	1.2 (1.1;1.3)	< 0.001	1.5 (1.2;1.8)
Serum PRO-C6 (ng/ml)	7 (5.7;8.6)	10.5 (8.1;14.9)	< 0.001	8.1 (6.8;12.2)
Serum C3M (ng/ml)	8 (6.9;9.3)	9 (7.2;11.2)	0.083	8.4 (7.3;9.7)
Urine PRO-C6 (ng/µmol)	0.2 (0.2;0.3)	0.2 (0.2;0.3)	0.010	0.2 (0.2;0.3)
Urine C3M (ng/µmol)	5.6 (3.9;7.4)	3.7 (2.4;5.7)	< 0.001	6.5 (3.9;7.5)

Data are in means (SD), medians (IQR) or n (%). RAAS: renin–angiotensin–aldosterone system; ARBs: angiotensin receptor blockers; CAN: cardiovascular autonomic neuropathy; PRO-C6: Collagen type VI (COL6); C3M III: Collagen type (COL3)

### Serum PRO-C6 and C3M

**Fig. 1:**
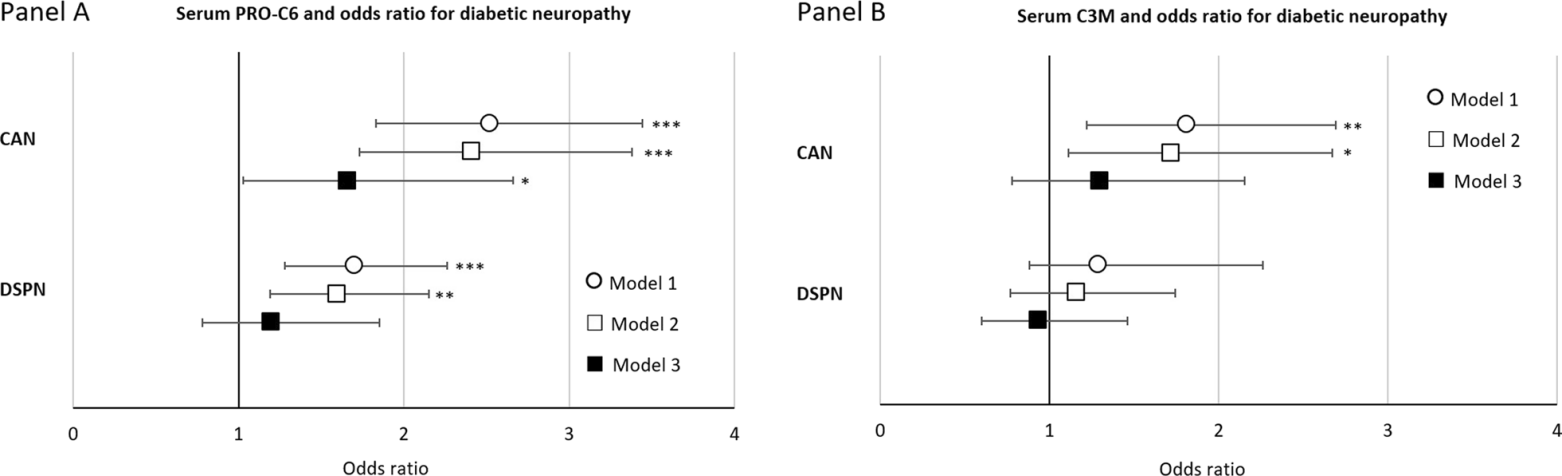
Odds ratios of CAN or DSPN by a 50% higher level of serum collagen markers. Odds ratios (95% confidence intervals) of logistic regression analyses of associations between serum PRO-C6 (panel **A**) and serum C3M (panel **B**) and CAN and DSPN. Odds ratios are presented as a 50% increase in determinants: Model 1 adjusted for age and sex, Model 2 further adjusted for diabetes duration, HbA_1c_, BMI, smoking, systolic blood pressure, LDL cholesterol and beta blocker treatment (only for CAN), Model 3 additionally adjusted for eGFR. CAN: cardiovascular autonomic neuropathy; DSPN: distal symmetrical polyneuropathy; ***p < 0.001, *p < 0.05

Logistic linear regression analyses showed that higher sPRO-C6 was associated with an increased odds ratio of having the CAN diagnosis in all levels of adjustments (Fig. 1, panel A).

In the fully adjusted models, a 50% higher sPRO-C6 was associated with an odds ratio of 1.66 (95% CI 1.03–2.66) [p = 0.035] for CAN (Table 2). Similar associations were seen for sPRO-C6 and DSPN. Here, higher levels of sPRO-C6 were associated with higher odds ratio for DSPN (Fig. 1, panel A). Statistical significance was retained in models 1 and 2. In model 2 a 50% higher level of sPRO-C6 was associated with an odd ratio of 1.60 (95% CI 1.19–2.15) [p = 0.001] for DSPN. After adjustment for eGFR in model 3 the odds ratio for DSPN was still above 1, but statistical significance was lost (Table 2).

**Table 2:** Odds ratios of CAN or DSPN by a 50% higher level of serum and urine collagen markers

	Model 1	Model 2	Model 3
*Odds ratio of CAN*			
Serum PRO-C6 (ng/ml)	2.44 (1.78;3.34) [< 0.001]	2.41 (1.73;3.38) [< 0.001]	1.66 (1.03;2.66) [0.035]
Serum C3M (ng/ml)	1.78 (1.19;2.66) [0.004]	1.73 (1.11;2.67) [0.012]	1.30 (0.78;2.15) [0.312]
Urine PRO-C6 (ng/µmol)	1.74 (1.06;2.88) [0.018]	1.66 (0.96;2.89) [0.049]	0.86 (0.48;1.54) [0.615]
Urine C3M (ng/µmol)	0.56 (0.42;0.73) [< 0.001]	0.57 (0.42;0.76) [< 0.001]	0.85 (0.60;1.21) [0.380]
*Odds ratio of DSPN*			
Serum PRO-C6 (ng/ml)	1.70 (1.28;2.26) [< 0.001]	1.60 (1.19;2.15) [0.001]	1.20 (0.78;1.84) [0.404]
Serum C3M (ng/ml)	1.29 (0.88;1.90) [0.192]	1.16 (0.77;1.74) [0.475]	0.94 (0.60;1.46) [0.768]
Urine PRO-C6 (ng/µmol)	1.22 (0.76;1.04) [0.415]	1.02 (0.62;1.70) [0.925]	0.69 (0.39;1.21) [0.195]
Urine C3M (ng/µmol)	0.81 (0.63;1.05) [0.108]	0.85 (0.65;1.10) [0.223]	1.17 (0.82;1.67) [0.373]

Estimates of linear logistic regression models presented as odds ratios (95% confidence intervals, P-values) of CAN or DSPN as a function of a 50% increase in determinants: Model 1 adjusted for age and sex, Model 2 further adjusted for diabetes duration, HbA_1c_, BMI, smoking, systolic blood pressure, LDL cholesterol and beta blocker treatment (only for CAN), Model 3 additionally adjusted for eGFR. CAN: cardiovascular autonomic neuropathy; DSPN: distal symmetrical polyneuropathy; PRO-C6: Collagen type VI (COL6); C3M III: Collagen type (COL3)

Higher levels of sC3M were associated with increased odds ratio of CAN (Fig. 1, panel B). A 50% higher level of sC3M was associated with a higher odds ratio of CAN in model 1 and 2. In model 2, the odds ratio was 1.73 (95% CI 1.11–2.67) [p = 0.012]. Significance was lost after additional adjustment for eGFR, still with an odds ratio above 1 (Table 2). Higher levels of sC3M trended toward a higher odds ratio of DSPN in all models of adjustments, but associations were not statically significant.

### Urine PRO-C6 and C3M

**Fig. 2:**
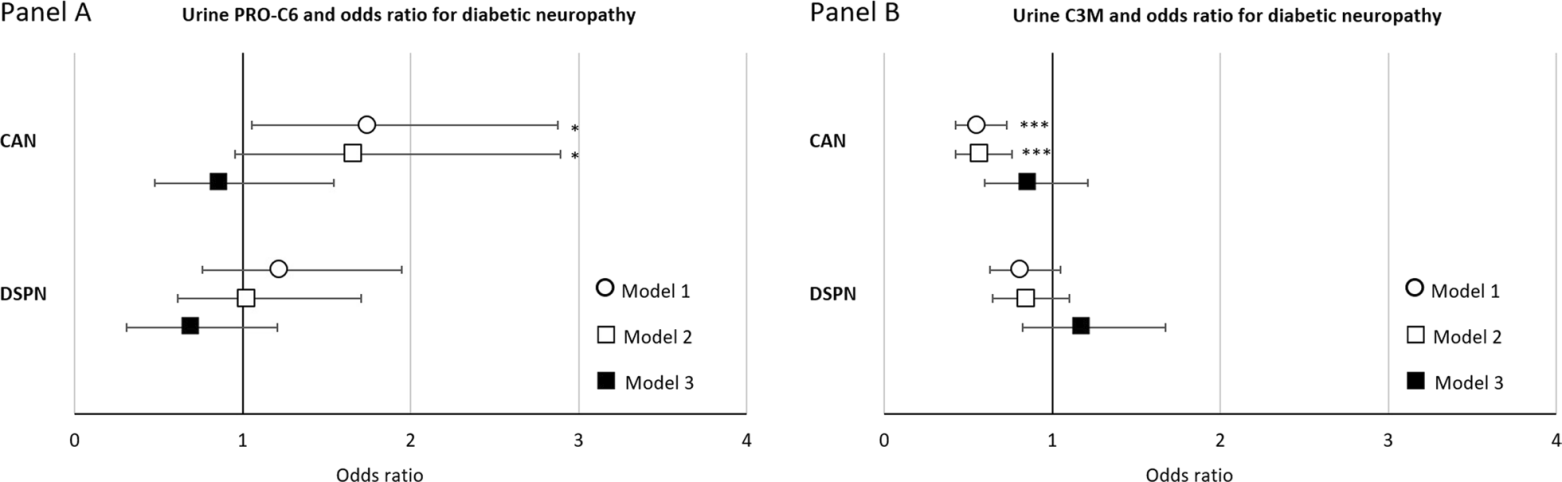
Odds ratios of CAN or DSPN by a 50% higher level of urine collagen markers. Odds ratios (95% confidence intervals) of logistic regression analyses of associations between urine PRO-C6 (panel **A**) and urine C3M (panel **B**). Odds ratios are presented as a 50% increase in determinants: Model 1 adjusted for age and sex, Model 2 further adjusted for diabetes duration, HbA_1c_, BMI, smoking, systolic blood pressure, LDL cholesterol and beta blocker treatment (only for CAN), Model 3 additionally adjusted for eGFR. CAN: cardiovascular autonomic neuropathy; DSPN: distal symmetrical polyneuropathy; ***p < 0.001, **p < 0.01, *p < 0.05

Higher levels of uPRO-C6 were associated with increased odds of CAN in model 1 with an odd ratio of 1.66 (95% CI 1.03;2.66) [p = 0.035]. The association remained significant in model 2 (p = 0.049). Associations were lost after additional adjustments for eGFR. UPRO-C6 was not associated with DSPN (Fig. 2, panel A, and Table 2).

Higher uC3M was significantly associated with a lower odds ratio of CAN in model 1 and 2, but not in model 3. UC3M was not associated with DSPN (Fig. 2, panel B, and Table 2).

### Cardiovascular autonomic reflex test (CARTs) and serum PRO-C6 and C3M

**Fig. 3:**
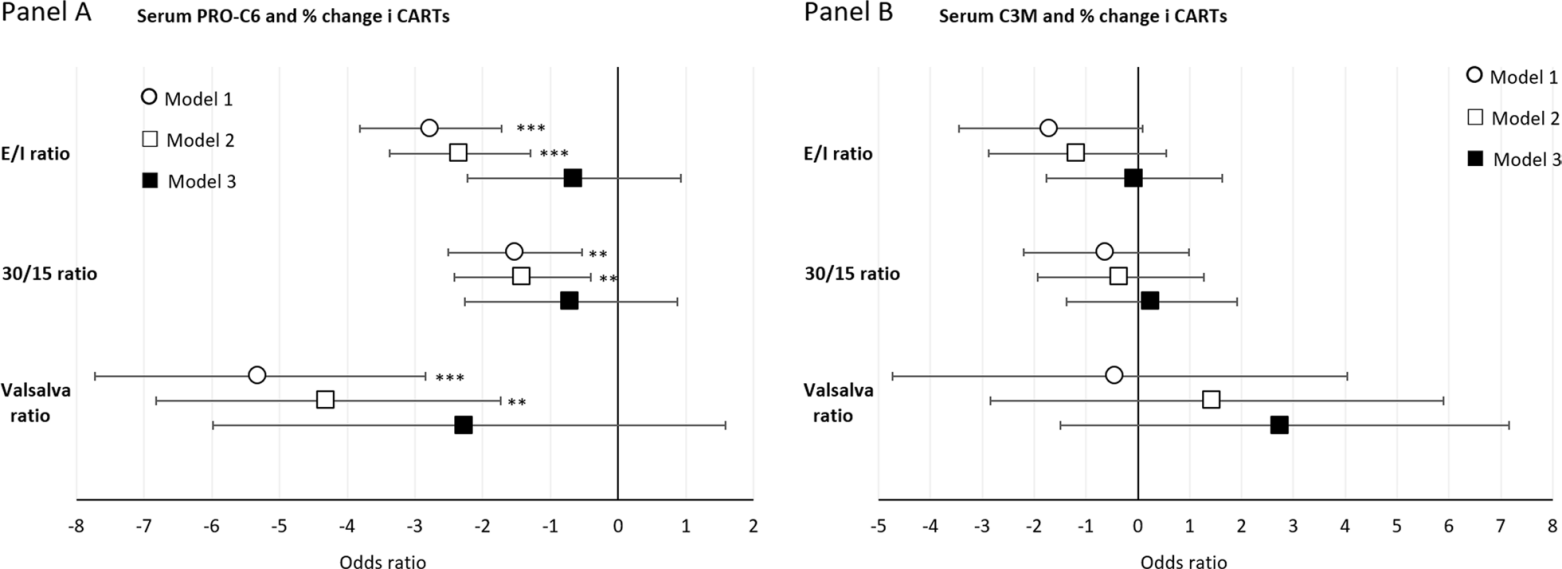
% change of CARTs by a 50% higher level of serum collagen markers. % change (95% confidence intervals) of linear regression analyses of associations between serum PRO-C6 (panel **A**) and urine C3M (panel **B**). % change is presented as a 50% increase in determinants: Model 1 adjusted for age and sex, Model 2 further adjusted for diabetes duration, HbA_1c_, BMI, smoking, systolic blood pressure, LDL cholesterol and beta blocker treatment (only for CAN), Model 3 additionally adjusted for eGFR. CAN: cardiovascular autonomic neuropathy; DSPN: distal symmetrical polyneuropathy; ***p < 0.001, **p < 0.01

Regression analyses of association between serum collagen markers and cardiovascular autonomic reflex test (CARTs) show that higher levels of sPRO-C6 were associated with lower values of all three CARTs (Fig. 3, Table 3). Theses associations lost significance but retained directions for all CARTs when models were adjusted for eGFR. Most pronounced associations were found for the Valsalva ratio where a 50% higher level of sPRO-C6 were associated with a 4.31% (95% CI 1.74%; 6.82%) [p = 0.001] in model 2. Associations were less pronounced for the E/I ratio and even less so for the 30/15 ratio where a 50% higher level of sPRO-C6 were associated with a 1.42% lower (95% CI 0.41%; 2.42%) [p = 0.006] estimate in model 2.

**Table 3:** % changes in CARTs by a 50% higher level of serum and urine collagen markers

	Model 1	Model 2	Model 3
*E/I ratio*			
Serum PRO-C6 (ng/ml)	− 2.77 (− 3.83; − 1.72) [< 0.001]	− 2.34 (− 3.38; − 1.30) [< 0.001]	− 0,66 (− 2.23;0.93) [0.411]
Serum C3M (ng/ml)	− 1.70 (− 3.45;20.08) [0.063]	− 1.18 (− 2.87;0.55) [0.181]	− 0.08 (0.176;1.63) [0.929]
Urine PRO-C6 (ng/µmol)	− 2.05 (− 4.03; − 0.03) [0.048]	− 1.71 (− 3.63;0.230) [0.086]	0.32 (− 1.73;2.42) [0.761]
Urine C3M (ng/µmol)	2.42 (1.37;3.49) [< 0.001]	1.97 (0.95;3.01) [0.002]	0.82 (− 0.39;2.04) [0.187]
*30/15 ratio*			
Serum PRO-C6 (ng/ml)	1.02 (0.04; 2.01) [0.043]	− 1.42 (− 2.42; − 0.41) [0.006]	− 0.71 (− 2.27;0.87) [0.378]
Serum C3M (ng/ml)	− 0.63 (− 2.21;0.98) [0.442]	− 0.35 (− 1.94;127) [0.670]	0.25 (− 1.37;1.90) [0.760]
Urine PRO-C6 (ng/µmol)	− 0.40 (− 2.26;1.49) [0.675]	− 0.32 (− 2.18;1.58) [0.741]	0.89 (− 1.16;2.99) [0.399]
Urine C3M (ng/µmol)	0.81 (0.63;1.05) [0.108]	0.86 (− 0.14;1.86) [0.093]	0.17 (− 1.03;1.39) [0.781]
*Valsalva ratio*			
Serum PRO-C6 (ng/ml)	− 5.32 (− 7.73; − 2.85) [< 0.001]	− 4.31 (− 6.82; − 1.74) [0.001]	1.20 (0.78;1.84) [0.404]
Serum C3M (ng/ml)	− 0.46 (− 4.74;4.04) [0.843]	1.43 (− 2.85;5.90) [0.518]	2.74 (− 1.50;7.16) [0.209]
Urine PRO-C6 (ng/µmol)	− 6.49 (− 10.47; − 2.33) [0.003]	− 5.26 (− 9.27; − 1.08) [0.015]	− 2.17 (− 6.76;2.67) [0.373]
Urine C3M (ng/µmol)	4.78 (2.07;7.55) [< 0.001]	4.20 (1.53;6.95) [0.002]	2.31 (− 0.74;5.44) [0.141]

Estimates of linear regression models presented as % changes (95% confidence intervals, P-values) for CARTs as a function of a 50% increase in determinants: Model 1 adjusted for age and sex, Model 2 further adjusted for diabetes duration, HbA_1c_, BMI, smoking, systolic blood pressure, LDL cholesterol and beta blocker treatment (only for CAN), Model 3 additionally adjusted for eGFR. CARTs: cardiovascular autonomic reflex tests; PRO-C6: Collagen type VI (COL6); C3M III: Collagen type III (COL3)

Regression models showed no statistically significant associations between sC3M and levels of CARTs (Fig. 3, Table 3).

### Cardiovascular autonomic reflex test (CARTs) and urine PRO-C6 and C3M

**Fig. 4:**
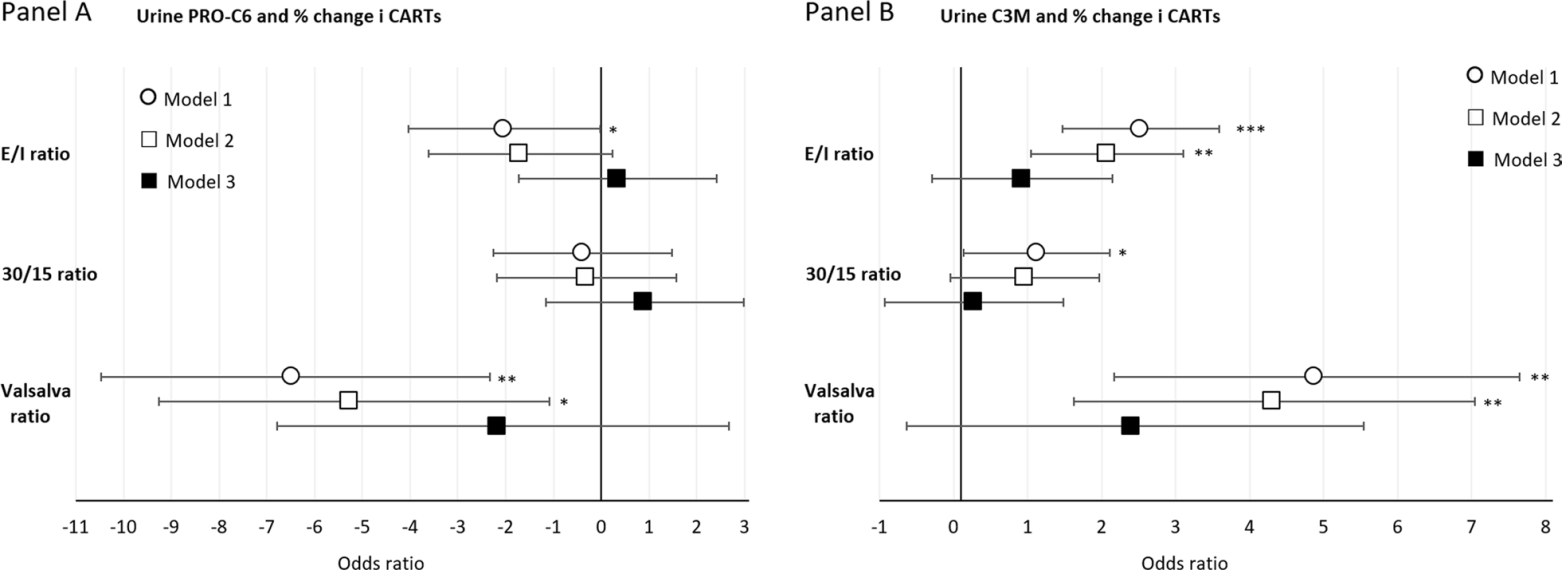
% change of CARTs by a 50% higher level of urine collagen markers. % change (95% confidence intervals) of linear regression analyses of associations between urine PRO-C6 (Panel A) and urine C3M (Panel B). % change is presented as a 50% increase in determinants: Model 1 adjusted for age and sex, Model 2 further adjusted for diabetes duration, HbA_1c_, BMI, smoking, systolic blood pressure, LDL cholesterol and beta blocker treatment (only for CAN), Model 3 additionally adjusted for eGFR. CAN: cardiovascular autonomic neuropathy; DSPN: distal symmetrical polyneuropathy; ***p < 0.001, **p < 0.01

Regression analyses of association between urine collagen markers and cardiovascular autonomic reflex test (CARTs) show that higher levels of uPRO-C6 were associated with lower values of the E/I-ratio (only statistically associated in model 1) and the Valsalva ratio (only statistically associated in model 1 and 2) (Fig. 4, Table 3). Most pronounced associations were found for the Valsalva ratio where a 50% higher level of uPRO-C6 were associated with a 5.26% lower (95% CI 1.08%; 9.27%) [p = 0.015] estimate in model 2.

Regression estimates showed that higher levels of sC3M were associated with higher values of all CARTs. However, for the 30/15 ratio only in model 1. In addition, significance was lost after adjustment for eGFR for the remaining CARTs. Most pronounced associations were found for the Valsalva ratio where a 50% higher level of uC3M were associated with a 4.20% higher (95% CI 1.53%; 6.95%) [p = 0.015] estimate in model 2.

### Collagen turnover markers and the CAN diagnosis in patients with normal kidney function.

Sensitive analyses in patient with eGFR ≥ 90 (n = 94) showed that higher sPRO-C6 was associated with higher odds ratio of CAN. No other significant associations were seen (Table 4).

**Table 4:** Odds ratios of CAN by a 50% higher level of serum and urine collagen markers

	Model 1	Model 2	Model 3
*Odds ratio of CAN*			
Serum PRO-C6 (ng/ml)	2.13 (1.05;4.30) [0.032]	2.68 (1.12;6.44) [0.02]	2.53 (1.06;6.04) [0.030]
Serum C3M (ng/ml)	1.66 (0;77;3.55) [0.193]	2.08 (0.82;5.25) [0.125]	1.92 (0.74;4.97) [0.185]
Urine PRO-C6 (ng/µmol)	0.12 (0.00;41.90) [0.459]	0.01 (0.00;143.05) [0.297]	0.00 (0.00;50.35) [0.197]
Urine C3M (ng/µmol)	0.98 (0.43;1.16) [0.940]	0.83 (0.46;1.50) [0.534]	0.86 (0.48;1.57) [0.632]

Estimates of linear logistic regression models presented as odds ratios (95% confidence intervals, P-values) of CAN or DSPN as a function of a 50% increase in determinants: Model 1 adjusted for age and sex, Model 2 further adjusted for diabetes duration, HbA_1c_, BMI, smoking, systolic blood pressure, LDL cholesterol and beta blocker treatment (only for CAN), Model 3 additionally adjusted for eGFR. CAN: cardiovascular autonomic neuropathy; DSPN: distal symmetrical polyneuropathy; PRO-C6: Collagen type VI (COL6) and C3M III: Collagen type (COL3)

## Discussion

### Collagen markers and CAN

In this cross-sectional study where 259 people with T1D had a CAN estimation and with a substantial proportion diagnosed with CAN (29.3%), we found a significant and clinically relevant association between higher levels of sPRO-C6 and the odds of having CAN even after adjustment for relevant confounders. Analyses of associations between collagen turnover markers and continuous values of the three CARTs used to diagnose CAN showed that higher levels of sPRO-C6 were associated with worse results in the autonomic tests. Levels of sC3M were not associated with CARTs. This demonstrates that higher sPRO-C6 may affect both the parasympathetic and sympathetic nervous system, as the E/I ratio mainly is marker of by parasympathetic activity, the 30/15 ratio is a mixed parasympathetic and sympathetic measure and the Valsalva ratio in a mixed sympathetic and parasympathetic measure [29].

The role of COL6 in the pathogenesis of fibrosis has been studied thoroughly. COL6 is a core structural component of the extracellular matrix of numerous tissues including nerves [30]. In addition, COL6 plays a central role in cellular functions such as apoptosis, cell proliferation and autophagy [31]. Of note, the regulation of COL6 has been suggested to be independent of other components of the extracellular matrix such as collagen type I and COL3 [32]. Specifically for nerves, COL6 is an important factor in Schwann cell differentiation [33]. A marked upregulation of COL6 is seen in animal peripheral nerve injury models [34]. Also in animal studies, loss of COL6 was associated with reduced nerve conduction and loss of motor control [35]. Thus, COL6 has an important role in nerve integrity and function. Conversely, as sPRO-C6 has been associated with several complications such as nephropathy and vascular disease in diabetes [14–16]. Also, endotrophin, the bioactive byproduct of COL6 which is measured as sPRO-C6, have been shown to have proinflammatory and profibrotic properties [23] which in itself may induce nerve dysfunction. Whether higher sPRO-C6 could be a sign of counter regulatory mechanisms to deleterious changes in nervous tissue or a cause of these damages is unclear. Our data demonstrate that COL6 turnover assessed by sPRO-C6 concentrations is associated with presence of CAN in T1D. We hypothesize that the increased levels of sPRO-C6 may reflect an increased level of fibrosis. It remains to be investigated if autonomic fibers from people with CAN display signs of increased fibrosis, such as seen for diabetic peripheral nerves [20].

Associations between uPRO-C6 and CAN showed similar patterns as for the serum markers, but less pronounced and with no associations after adjustment for eGFR. Associations between uPRO-C6 and CARTs suggested that the association to CAN may be mediated by effects on both the parasynaptic and sympathetic nervous system as inverse associations were mainly seen between uPRO-C6 and the Valsalva ratio. Associations were lost after eGFR adjustments. This attenuation of associations by eGFR adjustment for both serum and urine markers could be due to increased collagen turnover in the kidney with worse kidney function followed by increased spill-over to blood and urine of PRO-C6 originated in the kidney. Thus, kidney function (eGFR) could confound associations and explain loss of associations for urine analyses. In the present study design, it is not possible to assess the tissue specificity of PRO-C6. Thus, it remains unclear if kidney function is a true confounder for the analyses.

Similar association was seen for sC3M as for sPRO-C6. However, these associations lost significance after adjustment for eGFR. COL3 and its turnover products have not been investigated as intensively as COL6. However, as with COL6, COL3 is a component of the extracellular matrix of peripheral nerves [19, 36] and has been suggested to impair nerve regeneration after nerve injury [37]. Again, the study design does not allow to assess if kidney function is a true confounder of associations. Levels of C3M have been shown to allow for discrimination between people with and without kidney disease [38]. However, this does not necessarily prove that kidney function confounds the association between C3M and CAN. Kidney dysfunction is a concomitant complication to neuropathy [8], and therefore adjusting for kidney function may be a case of overadjustment.

UC3M showed an inverse association with the CAN diagnosis, but only in model 1 and 2. These results mirror the findings seen in the serum analyses. Higher levels of uC3M were associated with better/ higher values of both the E/I ratio and Valsalva ratio in model 1 and 2. The loss of associations after adjustment for kidney function could suggest could be due to processes in the kidney.

In the subset of participants with eGFR ≥ 90 only associations between sPRO-C6 and CAN were significantly associated. Here, higher sPRO-C6 was associated with higher odds ratio of CAN. Suggesting, that at least for sPRO-C6 findings were not mediated by kidney function.

### Collagen markers and DSPN

In the study cohort of 300 participants, 43% had DSPN. Associations between s- and uPRO-C6 and s- and u C3M and the odds of having DSPN showed similar directional patterns as for CAN. Here, higher levels of sPRO-C6 were associated with a higher odds ratio of DSPN, but not after adjustment for eGFR. UPRO-C6 showed a trend toward a positive association to DSPN when adjusted for relevant confounders, but not when including kidney function in the adjusted analyses. These findings indicate that COL6 formation is associated with the presence of late peripheral large fiber neuropathy, as seen for CAN. Similar non-significant trends were seen for sC3M and an inverse non-significant trend for uC3M and DSPN. This inverse and unexpected association remains to be clarified.

## Conclusions

In concert, our results show that a higher collagen turnover is associated with presence of CAN in T1D and possibly also DSPN. To our knowledge an association between CAN and markers of collagen turnover has never been described. Here we demonstrate that higher levels of sPRO-C6 and sC3M are associated with a higher odds ratio of CAN. In addition, higher levels of sPRO-C6 were associated with DSPN. Higher levels of uPRO-C6 were associated with higher odds ratio of CAN. However, additional associations between urine markers of collagen turnover and neuropathy outcomes were not demonstrated, except for an inverse association between uPRO-C6 and CAN in models not adjusted for kidney function.

Whether increased collagen turnover is a risk factor of future incident CAN or DSPN, as seen for other diabetic complications, such as cardiovascular disease and nephropathy [14–16] remains to be explored. Our data could warrant interventional trials investigating the efficacy of drugs with anti-fibrotic properties such as mineralocorticoid receptor antagonists on diabetic neuropathy [39], as these drugs have shown to improve CAN indices in other populations with congestive heart failure [40] and kidney disease [41]. However, this suggested treatment implies a causal relationship between fibrosis and development of neuropathy. The cross-sectional design of the present study does not allow for conclusions on causality.

## Strengths

The strength of this study is that it is the first to investigate the association between CAN and DSPN and collagen turnover in individuals with T1D. Analyses were done on a substantial number of participants.

## Limitations

The markers of collagen turnover investigated in this study are not tissue specific. Therefore, associations may be confounded by mechanisms in non-neuronal tissue that could be concomitant to neuropathy.

In the present study a crude DSPN cut-off of vibration sensations threshold VPT of ≥ 25 V has used. According to the Toronto consensus criteria using such a method can only diagnose possible DSPN. Adherence to all suggested measuring modalities of the Toronto criteria could have improved the accuracy of the diagnosis. Also, using age- and height adjusted VPT threshold values could have allowed for a more correct DSPN diagnosis. However, the VPT of ≥ 25 threshold was applied as it is a threshold for severe DSPN and possibly more comparable to the CAN diagnosis in the degrees of neuropathy severity.

Beta blocker use was not a criterion of exclusion in the study. This may have led to a risk of misclassification of participants in study groups of CAN or no CAN. This was done to increase n and statistical power. To ameliorate the consequences of this possible misclassification analyses were adjusted for beta-blocker use.

Orthostatic blood pressure measures for orthostatic hypotension (OH) were not available in the study. Patients having OH could be considered as having late CAN when other CARTs are affected. Having these measures could have influenced the prevalence of CAN in the study.

The cross-sectional nature of the study does not allow for conclusions made on causality. Our findings may be caused by residual confounding. In theory our findings may be a result of reverse causation in the sense that autonomic dysfunction could elicit changes in collagen turnover. However, to our knowledge evidence of such an association has not been published. Participants with no CAN estimation were marginally older and had longer diabetes duration compared to those with sufficient CARTs performed to assess the presence of the CAN diagnosis. Persons without CAN estimation may have had worse autonomic function if tests were possible. Therefore, it is possible that we miss out on the more extreme values of autonomic dysfunction where associations may have been different. Results may not be generalizable to a broad diabetes population as participants were not randomly recruited from the outpatient clinic. Also, our finding cannot be extrapolated to younger patients with shorter diabetes duration. Participants recruited as a follow-up of a case–control study and therefor may represent two extremes of diabetic microvascular complications, e.g. people with and without albuminuria.

## Data Availability

The datasets generated during and/or analyzed during the current study are not publicly available due to restrictions by the Danish data protection agency. But are available from the corresponding author upon reasonable request.

## Abbreviations

BMI: Body mass index
CAN: Cardiovascular autonomic neuropathy
CARTS: Cardiovascular autonomic reflex tests
COL3: Collagen type III
COL6: Collagen type VI
eGFR: Estimated glomerular filtration ratio
SDNN: Standard deviation of normal-to-normal intervals
T1D: Type 1 diabetes
